# Focused analysis of RNFL decay in glaucomatous eyes using circular statistics on high-resolution OCT data

**DOI:** 10.1371/journal.pone.0292915

**Published:** 2023-10-18

**Authors:** Md. Hasnat Ali, Meghana Ray, S. Rao Jammalamadaka, Sirisha Senthil, M. B. Srinivas, Saumyadipta Pyne

**Affiliations:** 1 L. V. Prasad Eye Institute, Hyderabad, Telangana, India; 2 EEE Department, BITS Pilani Hyderabad Campus, Hyderabad, Telangana, India; 3 Indian Health Outcomes, Public Health and Economics Research (IHOPE) Center, Hyderabad, Telangana, India; 4 Health Analytics Network, Pittsburgh, PA, United States of America; 5 HEED Lab, LLC, Rockville, MD, United States of America; 6 Department of Statistics and Applied Probability, University of California, Santa Barbara, Santa Barbara, CA, United States of America; 7 EEE Department, BITS Pilani Dubai Campus, Dubai, UAE; The University of Iowa, UNITED STATES

## Abstract

We generated Optical Coherence Tomography (OCT) data of much higher resolution than usual on retinal nerve fiber layer (RNFL) thickness of a given eye. These consist of measurements made at hundreds of angular-points defined on a circular coordinate system. Traditional analysis of OCT RNFL data does not utilize insightful characteristics such as its circularity and granularity for common downstream applications. To address this, we present a new circular statistical framework that defines an Angular Decay function and thereby provides a directionally precise representation of an eye with attention to patterns of focused RNFL loss. By applying to a clinical cohort of Asian Indian eyes, the generated circular data were modeled with a finite mixture of von Mises distributions, which led to an unsupervised identification in different age-groups of recurrent clusters of glaucomatous eyes with distinct directional signatures of RNFL decay. New indices of global and local RNFL loss were computed for comparing the structural differences between these glaucoma clusters across the age-groups and improving classification. Further, we built a catalog of directionally precise statistical distributions of RNFL thickness for the said population of normal eyes as stratified by their age and optic disc size.

## Introduction

Progressive optic neuropathies such as glaucoma can cause irreversible blindness, especially when left untreated or diagnosed late. Glaucoma is a chronic ophthalmic disorder characterized by irreversible damage of ganglion cell and retinal nerve fiber layer (RNFL), progressive neuroretinal rim (NRR) thinning, and excavation of the optic nerve head (ONH) [1]. It is the second-leading cause of blindness worldwide [2]. In 2020, an estimated 80 million individuals worldwide had glaucoma and this number is expected to increase to over 111 million by 2040 [3, 4]. While early detection and management are essential for slowing the progression of glaucoma, substantial retinal ganglion cell loss could occur before the functional visual field loss from glaucoma is detected [5]. Standard automated perimetry (SAP) provides valuable information about the stage of glaucoma, but it is not useful for detecting small changes, particularly in early glaucoma. More than 30% of ganglion cell loss is needed before SAP shows the visual field loss [6].

The platform of spectral-domain optical coherence tomography (SD-OCT) provides excellent glaucoma-diagnostic performance and reproducibility [7, 8]. SD-OCT is a non-invasive scanning technology that provides precise, quantitative measurements of the retinal nerve fiver layer (RNFL), optic nerve head (ONH) parameters, neuro-retinal rim (NRR) area, and macular thickness–objective evaluation of these parameters have been used to identify glaucomatous structural damage. The RNFL data of the present study are generated with the Cirrus HD-OCT platform (software version 9.0.0.281; Carl Zeiss Meditec, Dublin, CA, USA) that uses Optic Disc Cube 200x200 protocol to scan the ONH and peripapillary area through a 6 mm square grid, which consists of 200 horizontal linear B-scans and each composed of 200 A-scans.

The most common technique to evaluate RNFL loss from OCT data is peripapillary RNFL measurement based on the calculation circle. Schuman et al. [9] set the scan circle to an arbitrary 3.4 mm diameter (S1A Fig). Subsequently, a 3.46 mm scan circle was set as a standard for glaucoma diagnostic, and all OCT manufacturers adopted the technique. First, the Cirrus HD-OCT algorithm identifies the center of ONH and then automatically places a calculation circle of 3.46 mm diameter evenly around it. Then the system extracts from the data cube 256 A-scan samples and measures the RNFL thickness at each point along the path of calculation circle starting from the temporal quadrant at 9 o’clock in a clockwise direction for the right eye and for the left eye at 3 o’clock in a counter-clockwise manner in **T**emporal-**S**uperior-**N**asal-**I**nferior-**T**emporal (or TSNIT) order (S1B Fig).

Notably, the above approach makes the OCT platform, along with certain other biomedical scanning technologies, an ideal example of such generators of data-points that are indexed along multiple well-defined *directions*–here, at given angular positions equally-spaced around a central point. Unlike the analyses of “linear” data that reside as points on the real line or Euclidean spaces, directional data requires special and altogether different treatment. For instance, a particular direction in two-dimensional plane can be represented as a point on the circumference of a unit circle, or simply as an angle, but neither representation is unique, as both depend on the selection of some appropriate “zero-direction” as the reference starting point of measurement, as well as the sense of rotation, viz., clockwise or anti-clockwise. The unique properties of directional or circular data–for instance, if one wishes to compare or cluster such scans with a suitable distance measure–are appropriately addressed by the fields of directional and circular statistics [10–12].

Traditional OCT analysis often involves a simple division of the circle around ONH into 4 quadrants (of 90 degrees each) or 12 clock-hours (of 30 degrees each) to index and record measurements at these angular sections. Past studies on focused analysis of angular sections of OCT RNFL and NRR data have revealed interesting differences between healthy and glaucoma subjects [13]. In this study, we extend such approaches by allowing for analysis of data collected at arbitrarily higher angular resolution that divide the circle (comprising of a total of 360 degrees) into a much larger number (*N*) of fine sections of 360/*N* degrees each. While our analytical framework holds in general for any value of *N*, here, we generated a circular sequence of *N* = 256 data points, which measures RNFL thickness at every 1.41 degrees, for each OCT sample from a human eye. Such high-resolution directional measurements, generated via automated processing of standard SD-OCT images, are ideally suited for the application of new and insightful techniques from circular statistics for *directionally-focused* studies of OCT RNFL data, as we introduce in this study.

In addition to age, on the structural side, biological heterogeneity of ONH phenotypes, with or without any neuropathy, can also present challenges to clinical decision-making. Unsupervised learning of the heterogeneity of normative ONH phenotypes in a given population can provide key insights into the diversity of baselines that may exist for degenerative neuropathies, and thus, reduce subjectivity in diagnosis [14]. Such knowledge is particularly useful in glaucoma for which different ONH parameters play a combined role in early detection. For instance, in a non-glaucoma multiethnic cohort of Asian individuals, the inter-eye RNFL profile was found by OCT to be less symmetric in Malays and Indians than that in Chinese eyes [15]. Even the manufacturers of OCT technology noted racial differences in optic disc area, RNFL thickness, etc., when measured using their platform [16]. Thus, representation of diverse populations in OCT databases is a matter of great significance to glaucoma patients, e.g., more than 16 million in India of whom nearly 1.2 million could be blinded by the disease [4].

The present study addresses multiple important aims that include the design of an analytical framework that is capable of defining and using a *directional measure* of RNFL decay based on OCT data and then applying it for focused detection of structural characteristics in glaucomatous eyes from an Indian population. In this direction, our study makes several new contributions. First, we used a large clinical cohort of 3973 normal Indian eyes to generate high-resolution OCT RNFL data (as noted above) for each eye. Second, we built a catalog of directionally precise distributions of RNFL thickness for the population of normal eyes stratified by their age and optic disc size. At each angular-point on the circle, the RNFL distribution was computed using the empirical cumulative distribution function of the above OCT data. Third, we developed an analytical framework based on circular statistics that is ideally suited to OCT data analysis. It includes specifying a novel directional representation of RNFL decay of a given eye relative to its corresponding normal population. New indices were introduced to measure eye-specific both global as well as local RNFL decay. Fourth, we fit a finite mixture of von Mises distributions to identify for glaucomatous eyes two separate clusters of directional representations of RNFL decay, which were then characterized using their clinical covariates. Finally, we demonstrated the utility of these clusters in the refinement of glaucoma classification.

## Data and methods

### Data

All participants were selected from (i) LVPEI Glaucoma Epidemiology and Molecular Genetic Study (LVPEI-GLEAMS), a population-based study, and (ii) Longitudinal Glaucoma Evaluation Study (LOGES), a cross-sectional study. Both studies were conducted by the L.V. Prasad Eye Institute (LVPEI), Hyderabad, India [17]. Written informed consent was obtained from all subjects to participate in the study, and the LVPEI Institutional Ethics Committee reviewed and approved the methodologies for both the studies (LEC 08131 and LEC 11–252 for LVPEI-GLEAMS and LOGES respectively), which were conducted in strict adherence to the tenets of the Declaration of Helsinki. In the present study, we included a total of 3973 normal eyes (from 2222 healthy individuals) and 270 glaucomatous eyes (from 210 individuals). The clinical covariates of the cohort are shown in Table 1.

**Table 1:** Clinical covariates of the glaucomatous eyes.

Variable (unit)	mean	median	s.d.	min.	max.
RIM AREA (mm^2)	1.09	1.06	0.25	0.49	1.89
DISC AREA (mm^2)	2.28	2.22	0.59	1.23	4.69
AVERAGE CD RATIO	0.7	0.7	0.1	0.4	0.9
AVERAGE THICKNESS (μm)	86.84	87	9.62	54	112
VERTICAL CD RATIO	0.68	0.69	0.1	0.41	0.88
CUP VOLUME (mm^3)	0.52	0.34	0.49	0.04	3.38
DISK DIAMETER (mm)	1.62	1.6	0.22	1.1	2.4

(Abbreviations: CD = cup to disc, s.d. = standard deviation, min. = minimum, max. = maximum).

All participants underwent a comprehensive ophthalmic examination which included a detailed medical and systemic history, best-corrected visual acuity measurement, slit-lamp photographs (Topcon, Bauer Drive, Oakland, NJ), Goldmann applanation tonometry for intraocular pressure (Hagg-streit AT 900, Hagg-streit AG, Switzerland), gonioscopy with a Sussman four mirror gonioscope (Volk Optical Inc, Mentor, Ohio, USA), dilated fundus examination, central corneal thickness (CCT) assessment, Humphrey visual fields (HVF) with 24–2 Swedish Interactive threshold algorithm (SITA; Carl Zeiss Meditec Inc. Dublin, CA), digital optic disc photography and spectral-domain optical coherence tomography imaging with Cirrus HD-OCT (Carl Zeiss Meditec, Dublin, CA, USA) [18].

Visual fields (VF) were considered if false positive, false negative, and fixation losses less than 20%, and all the stereophotographs of the optic disc had good quality. The inclusion criteria were age ≥ 40 years, best-corrected visual acuity 20/40 or better, spherical equivalent (SE): ± 6D, good quality stereo optic disc photographs, and no media opacities (signal Strength ≥ 6). The exclusion criteria were intraocular surgery within the previous 6 months, any retinal (including macular) or neurologic diseases other than glaucoma that could confound the structural measurements with SD-OCT, RNFL thickness less than 30 μm, and the presence of blink and motion artifacts. The choice of circumpapillary RNFL thickness cutoff was based on previous studies on structural measurement floors by SD-OCT [19].

While healthy eyes were defined by the absence of anterior and posterior pathology, to determine a glaucomatous eye, beyond such measurements as VF and IOP, three glaucoma specialists independently evaluated its digital optic disc photograph. They were blinded of the other clinical findings and the other imaging outcomes of the individuals. Each disc photograph was defined as normal based on the absence of superior and inferior NRR thinning, rim notch, disc hemorrhage, and RNFL defect. Eyes were excluded from the study if there were any discrepancies among the specialists. Zeiss’ Cirrus HD-OCT software (version 9.0.0.281; Carl Zeiss Meditec, Dublin, CA, USA) uses the segmentation algorithm of the OCT software to identify the RNFL and measures its thickness on the circular peripapillary scan. For each eye (glaucomatous or normal) that was included in our analysis, the same algorithm was used for obtaining its high-resolution OCT RNFL thickness data at *N*( = 256) equally-spaced (360/*N* = 1.41 degrees apart) angular-points {*j*: *j* = 1,2,…*N*} in counterclockwise (TSNIT) direction around the circle (0–360 degrees). The high-resolution OCT RNFL thickness datasets for normal and glaucomatous eyes are provided in the S1 and S2 Tables respectively.

The eyes were first stratified according to different combinations of age and disc size. Three age categories: Age1 (40–49 years), Age2 (50–59 years), and Age3 (> = 60 years), and three optic disc size categories were used. These are Small (<1.6 mm^2^), Average (1.6–2.6 mm^2^), and Large (>2.6 mm^2^) discs. The cutoffs for disc size are derived from our prior work based on clinical evaluation which was later objectively quantified by HRT measurements [20]. Thus, over all 3×3 = 9 possible combinations of age and disc size, we determined for each eye *i* (normal or otherwise) exactly which group (call it *G*) it belongs to (denoted as *i*∈*G*).

### Angular Decay calculation

For each group *G*, and for each angular-point *j*, we take the set $R_{G,j}=\{RNFL(i_{N},j):\;normal\;eye\;i_{N}\in G\}$ of RNFL values at the angular point *j* for every normal eye *i*_*N*_∈*G*, and compute the empirical (cumulative) distribution function

$${eCDF}_{G,j}(x)=|{r\leq x:\;r\;\epsilon \;R}_{G,j}|/|R_{G,j}|.$$

Each function *eCDF*_*G*,*j*_ takes as input an RNFL thickness measurement *RNFL*(*i*_*G*_, *j*) of a glaucoma eye *i*_*G*_∈*G* at an angular-point *j*, and returns the corresponding quantile value, which lies in the range [0,1] where 0 and 1 correspond respectively to the lowest and the highest of all RNFL observations at the same angular-point *j* among the normal eyes that belong to *G*.

The Angular Quantile of RNFL data for an eye *i*∈*G* at an angular-point *j* is computed as

$$Angular\;Quantile(i,j)={eCDF}_{G,j}(RNFL(i,j)).$$

We note that the above quantile value provides a measure that is *relative* to all the RNFL observations made at a particular angular-point over the entire population of normal eyes of the corresponding group. Then the Angular Quantile is computed at each angular point *j*, which yields for an eye *i*∈*G* a circular sequence:

AngularQuantile(i)={AngularQuantile(i,j):j=1,…N}.

Thus, we define Angular Decay of an eye *i*∈*G* as the following circular sequence:

AngularDecay(i)={1−AngularQuantile(i,j):j=1,…N}.

Hence, for a given eye, at any angular-point, Angular Decay admits a value that lies in the range [0,1]. A higher value of Angular Decay in an eye corresponds to an OCT RNFL measurement that ranks relatively lower among the RNFL observations in the normal population.

### Directional representation of RNFL decay

Based on the normal RNFL quantile threshold *τ*, we characterize the Angular Decay values of a given eye in terms of those that are above versus below *τ*. Each distinct sequence of contiguous Angular Decay values exceeding *τ* is defined as a “petal”. Therefore, each petal (denoted by Π) represents an angular range that shows higher than normal decay. For a given threshold *τ*, we denote the number of such petals in an eye by *ν*_*τ*_. By fixing *τ*, say, to 0.75, we can drop the suffix in the above quantity (as well as those measurements defined below for each eye) and simply refer to it as *ν*.

In particular, we are interested in the “widest” petal (π*) of an eye, which is the largest contiguous sequence of Angular Decay values exceeding *τ*. We use an algorithm to traverse the circle counter-clockwise and at each successive angular-point *j* = 1,2,…*N* on the circle of eye *i* test whether *RNFL*(*i*,*j*) exceeds *τ*. This yields the starting point (*θ*_*s*_) and the end point (*θ*_*t*_) of each existing petal. The counter-clockwise circular interval [*θ*_*s*_, *θ*_*t*_] defines the angular range of a petal, and the widest petal, i.e., the one with the largest angular range, is identified as π*.

Since for each angular-point *j* in the range of π*, the Angular Decay value is known, we compute the *weighted circular mean* [21] of π* where the Angular Decay value at *j* is used as the weight *w*_*j*_ *ϵ* [0,1] for the angle *θ*_*j*_ at *j*. We denote this decay-weighted mean angle (of the largest contiguous region of the eye with above normal Angular Decay) by μ*, the quadrant specific inverse of the tangent (see Equation 1.3.5 on page 13 of [11])

$$\mu^{*}=\tan^{-1}(\sum_{j\epsilon \pi^{*}}w_{j}\;\sin\;\theta_{j}/\sum_{j\epsilon \pi^{*}}w_{j}\;\cos\;\theta_{j}).$$

The weighted mean angle allows μ* to provide a directional representation of an eye in terms of its regional concentration of relatively high RNFL loss. If no such region exists in a given eye for the chosen value of *τ*, then its μ* and π* are denoted by ‘NA’.

For unsupervised identification of clusters of eyes in a given collection with distinct directional signatures, the angular data (μ*) are fitted with a finite mixture of *K* univariate von Mises distributions $\sum_{k=1\ldots K}\alpha_{k}\;vM(\mu_{k},\kappa_{k})$ where *μ*_*k*_ and *κ*_*k*_ are respectively the (angular) location and the concentration parameters of the *k*^th^ component (cluster) of the above mixture whose non-negative proportions {*α*_*k*_: *k* = 1,…*K*} add up to 1. The optimal model is selected using the Bayesian Information Criterion (BIC) [22]. The absence of at least 2 distinct clusters in a given collection of eyes can be checked for this data with a test of circular unimodality [21]. The von Mises distribution *vM*(*μ*, *κ*) has the probability density function (pdf) $f(x|\mu ,\kappa )=\exp\left(\kappa \;\cos(x-\mu ))/2\pi I_{0}(\kappa \right)$, where *I*_0_(*κ*) is the modified Bessel function of the first kind of order 0 (11).

### Indices of local and global RNFL loss

We define 2 new indices of RNFL loss in a given eye. A measure of *local* RNFL loss in an eye is defined as the proportion of the size of the angular range of π* to the entire circle. This local loss index is denoted by λ*. Further, we also introduce a measure of *global* RNFL loss in an eye defined by the proportion of the total angular range of all ν petals in this eye to the entire circle. This global loss index is denoted by Λ.


$$\lambda^{*}=\{Number\;of\;points\;j\;in\;the\;angular\;range\;of\;\pi^{*}\}/N.$$

$$\Lambda =\sum_{\Pi =\Pi_{1}}^{\Pi_{\nu }}\left\{Number\;of\;points\;j\;in\;the\;angular\;range\;of\;\Pi \right\}/N.$$

Thus, both measures, λ*and Λ, take values in the range [0,1], where 0 and 1 represents respectively the minimal and the maximal RNFL loss in a given eye with respect to a chosen threshold *τ*. We note that 0≤λ*≤Λ≤1.

### Implementation

The above-mentioned framework, including all its functions for analysis and visualization, is implemented using the R platform. The empirical cumulative distributions and their 2-sample Kolmogorov-Smirnov tests were computed with the ecdf() and ks.test() functions respectively. The R package ‘movMF’ was used for fitting a finite mixture of univariate von Mises distributions with Expectation Maximization (EM) algorithm to the RNFL decay data represented as a set of angular measurements. For visualization of the Angular Decay in a given eye, our R program plots this function on a circular scale. Against a background blue circle of radius equal to a selected normal RNFL quantile threshold *τ*, the Angular Decay function is shown as a circular curve which appears in blue or red when it is below or above *τ* respectively. Each of the contiguous red sections is a separate petal Π and the widest petal π* with the largest angular range is shown in red (see Fig 3 for examples). The R package ‘ctree’ was used to build binary decision tree models for glaucomatous versus normal eye classification [23].

## Results

### Direction-specific catalog of normative RNFL thickness

**Fig 1:**
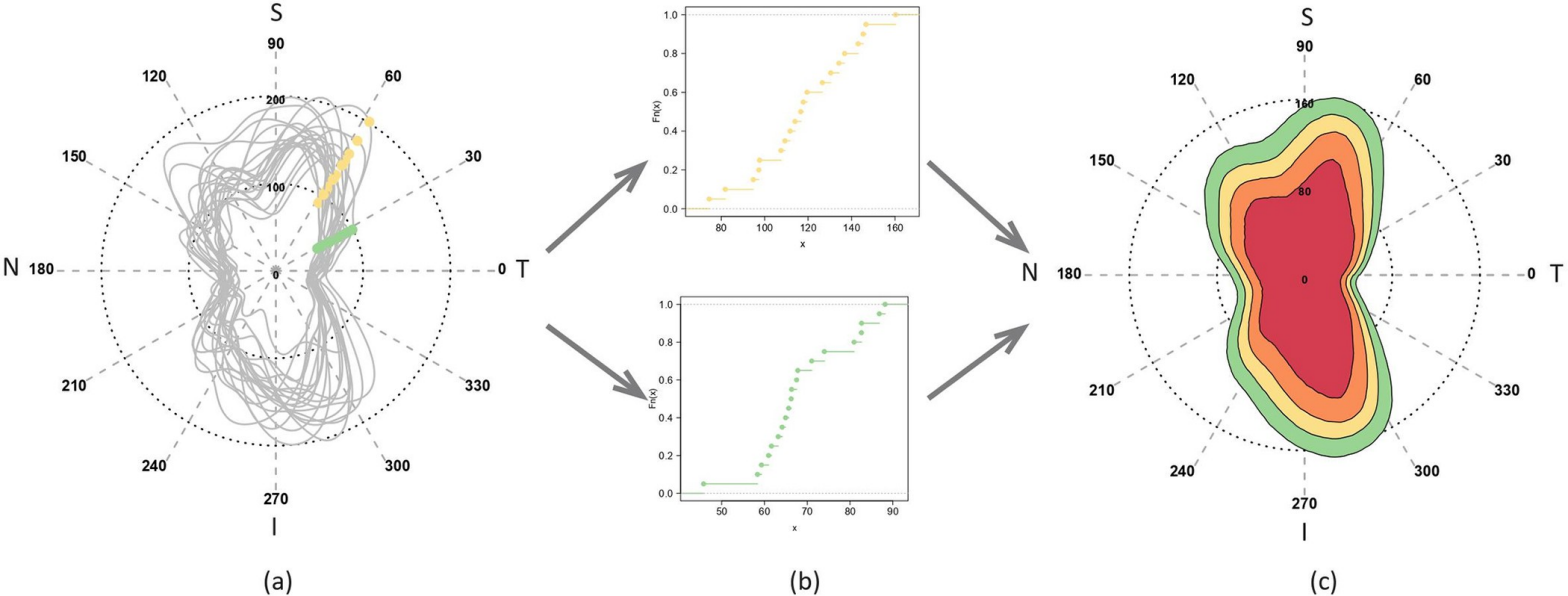
The circular OCT RNFL thickness data for normal eyes belonging to a group are overlaid and shown in plot (a). Two examples of angular-points are used to mark the OCT data as sets of green and orange points. Plot (b) shows the eCDF functions for these two OCT datasets. Plot (c) shows the ranges of normal RNFL thickness values for different Angular Quantiles. As examples, the concentric ranges for the 5^th^, 25^th^, 50^th^, and 75^th^ percentiles are shown with red, orange, yellow, and green contours respectively.

To construct a normative reference for the measure of RNFL loss in a given glaucomatous eye, we computed a population-based catalog of a total of 9×256 = 2304 direction-specific empirical distributions based on RNFL data from the 3985 normal eyes that belong to 9 groups (as defined in the Methods) and for *N* = 256 angular-points. The full catalog is given in S3 Table while the steps of computation are illustrated with an example in Fig 1. For a given group *G*, the RNFL thickness data of the normal eyes in *G* are plotted as circular curves in Fig 1(A). The RNFL measurements of these eyes at two arbitrarily fixed angular-points are marked by 2 sets of points in orange (*θ*_1_) and green (*θ*_2_). The empirical distribution functions of the sets *θ*_1_ and *θ*_2_ are shown in the corresponding colors in Fig 1(B). We cataloged such empirical distribution functions at every angular-point for each *G*. These yield the quantile of normative RNFL thickness at any angular-point. For instance, in Fig 1(C), for the 5^th^, 25^th^, 50^th^, and 75^th^ percentiles, the increasing ranges of normative RNFL thickness are shown as successive contour curves in red, yellow, orange, and green respectively. Thus, if the RNFL circular data of any new eye *i* is overlaid on this contour plot, it will reveal the precise extent of any global (based on magnitude) or local (based on magnitude *and* direction) RNFL loss relative to the normative RNFL thickness of the group that *i* belongs to.

### Direction-specific Angular Decay

**Fig 2:**
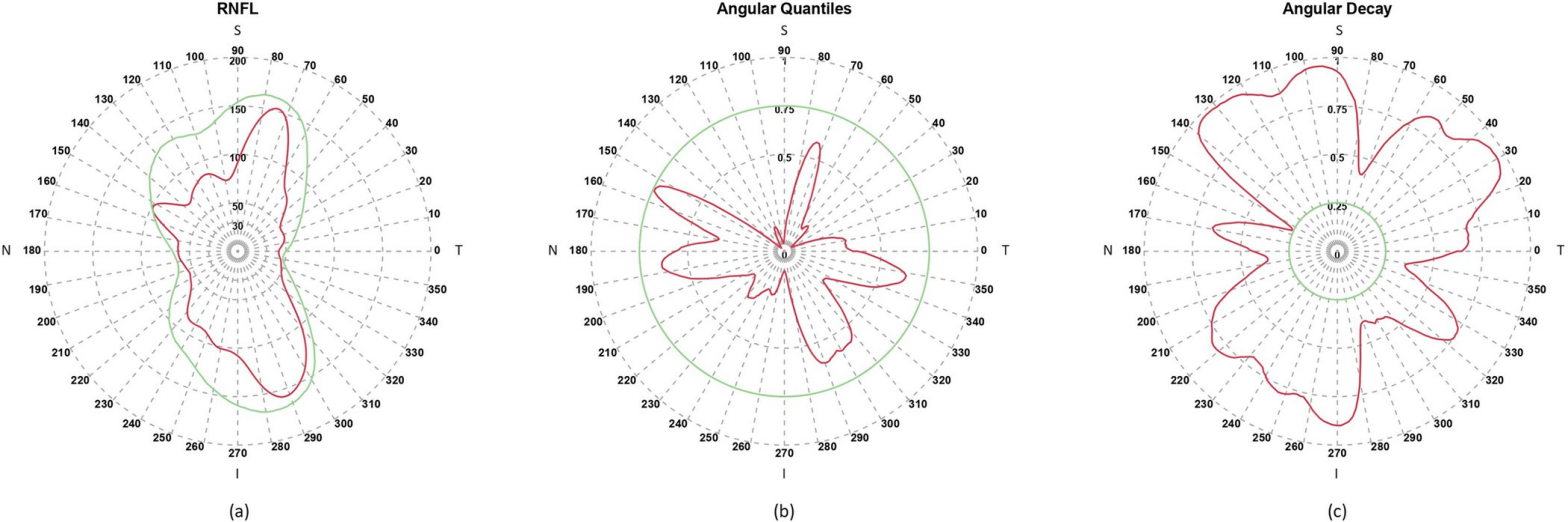
The circular OCT RNFL thickness data of a glaucomatous eye is shown as a red curve in plot (a). For reference, the 75^th^ percentile of normal RNFL thickness is shown with a green curve. The glaucomatous and normal RNFL data are converted to their respective Angular Quantiles (of range [0,1]) and shown as the corresponding red and green curves in plot (b). In (c), the complementary plot of (b) shows the RNFL Angular Decay (of range [0,1]) as red and green curves for the glaucomatous and normal cases respectively.

We calculated this as a circular function based on an eye *i*‘s high-resolution OCT RNFL thickness data at each angular-point *j*. Given the group *G* that *i* belongs to, and *j*, the corresponding distribution function from the catalog (described above) is used for calculating the precise quantile value of RNFL thickness of *i* at *j*. This is shown as the red curve in Fig 2(A). To focus on the last quartile (i.e., 75^th^-100^th^ percentile) where the RNFL loss is likely to be the most common, a green curve shows the normative RNFL thickness at quantile 0.75 for reference. The quantile values of each RNFL curve at every angular-point yield the corresponding Angular Quantile curves shown using the same colors in Fig 2(B). The exactly complementary curves (computed as *Angular Decay* = 1−*Angular Quantile*) produces the direction-specific values of Angular Decay of the eye *i* shown, again using the same colors, in Fig 2(C). Since the Angular Quantile function has the range [0,1], so does Angular Decay. The RNFL data, the corresponding Angular Quantile and Angular Decay functions are depicted as the red curves in Fig 2(A)–2(C) respectively, and due to an arbitrary (but real) eye that serves as an example.

### Contiguous regions of Angular Decay

**Fig 3:**
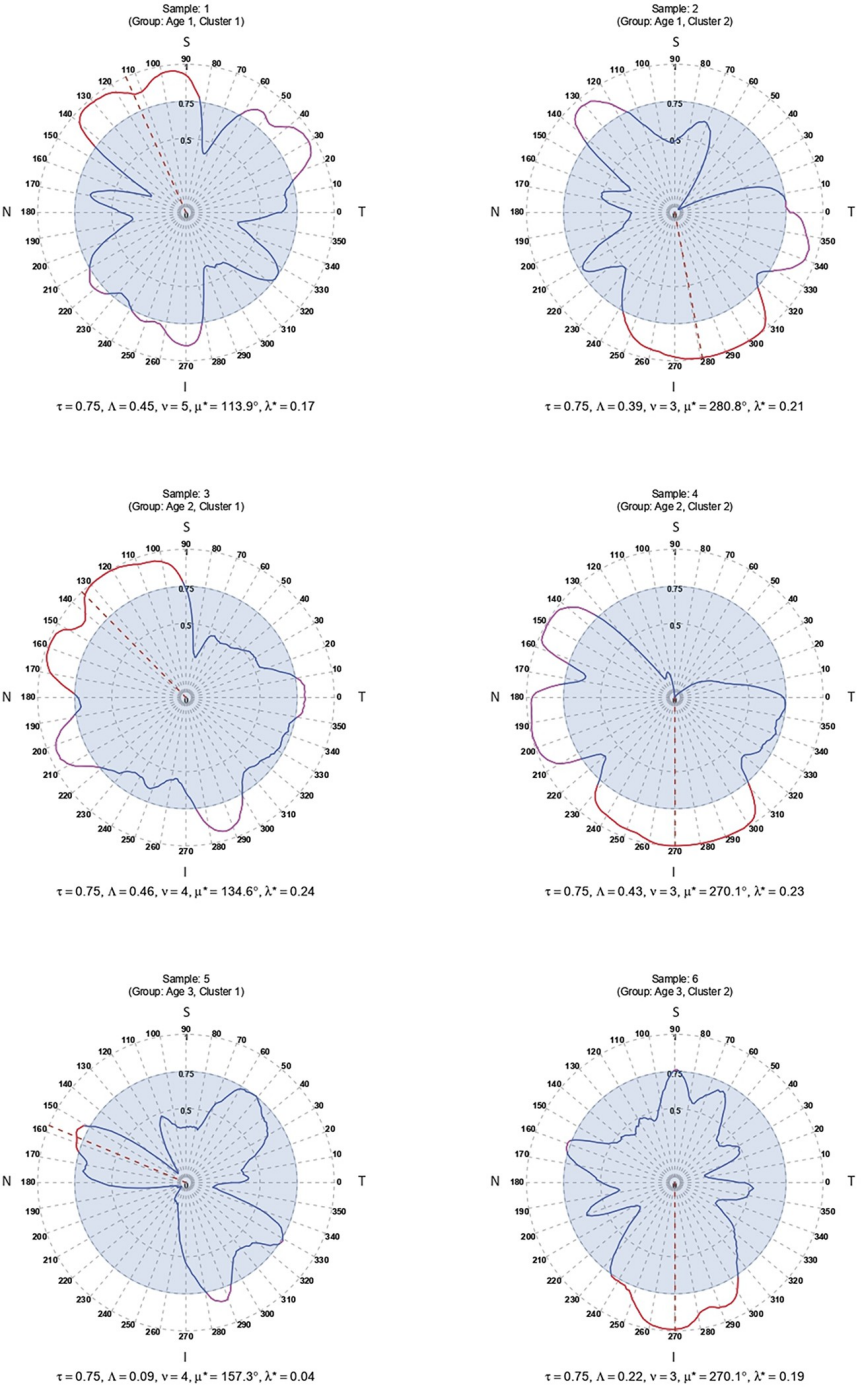
For 6 samples of glaucomatous eyes, the RNFL Angular Decay functions are plotted. Each plot contains a pale blue circle of radius equal to the threshold of decay τ set at 0.75. The petals lying above the pale blue circle are shown as pink curves. The petal with the largest angular range is marked with a red curve. The weighted circular mean angle of the red petal is shown with a dashed red line. Various indices computed for each eye are shown below the plot of each sample.

To identify locally distinctive features of an eye’s RNFL loss, we applied a circular tracing algorithm that uses a user-defined quantile threshold (*τ*) to demarcate the “petals” which are contiguous regions of Angular Decay values exceeding *τ*. Thus, we identified the petals for a given eye to compute insightful patterns of RNFL loss using 4 new indices. To illustrate, the Angular Decay functions of 6 glaucomatous eyes are shown in Fig 3 as examples. For each eye, the selected quantile threshold is shown as a pale blue circle of radius *τ*. The Decay function is shown as blue or pink curves depending on whether it takes values below or above *τ*. Each contiguous pink angular section represents a separate petal denoted by Π. In a given eye, for fixed *τ*, the total number of petals is denoted by *ν*; and the petal with the largest angular range is depicted as a red curve and denoted by π*.

### Angular representation of an eye’s RNFL decay

The systematic identification of π* allows us to define an angular representation of an eye in terms of its largest contiguous section of above normal RNFL decay. For this purpose, we compute the weighted circular mean of π*, which is the angle (denoted by μ*) around which the RNFL decay within the widest petal of the eye is concentrated. The weighting used in the calculation of μ* captures the contribution of the variations of the shape of π*, which results in a more precise directional representation of the decay than its unweighted counterpart. The angular value of μ* for each eye is shown as a dashed red line in the Angular Decay plots of Fig 3.

### Local and global RNFL loss

In addition to such directional information, we also quantified the proportion of decay that is described by π* as it represents the widest petal of an eye. Thus, we define a measure of *local loss* of RNFL of an eye, denoted by λ*, as the fraction of the full circle that is covered by the angular section corresponding to π*. Further, we introduce another index for measuring the *global loss* of RNFL for an eye, denoted by Λ, as the fraction of the full circle that is covered by the union of the angular sections corresponding to all the petals in an eye. In Fig 3, below each eye’s Angular Decay plot, the computed values of its different indices are shown.

### Circular clustering and clinical characterization

**Fig 4:**
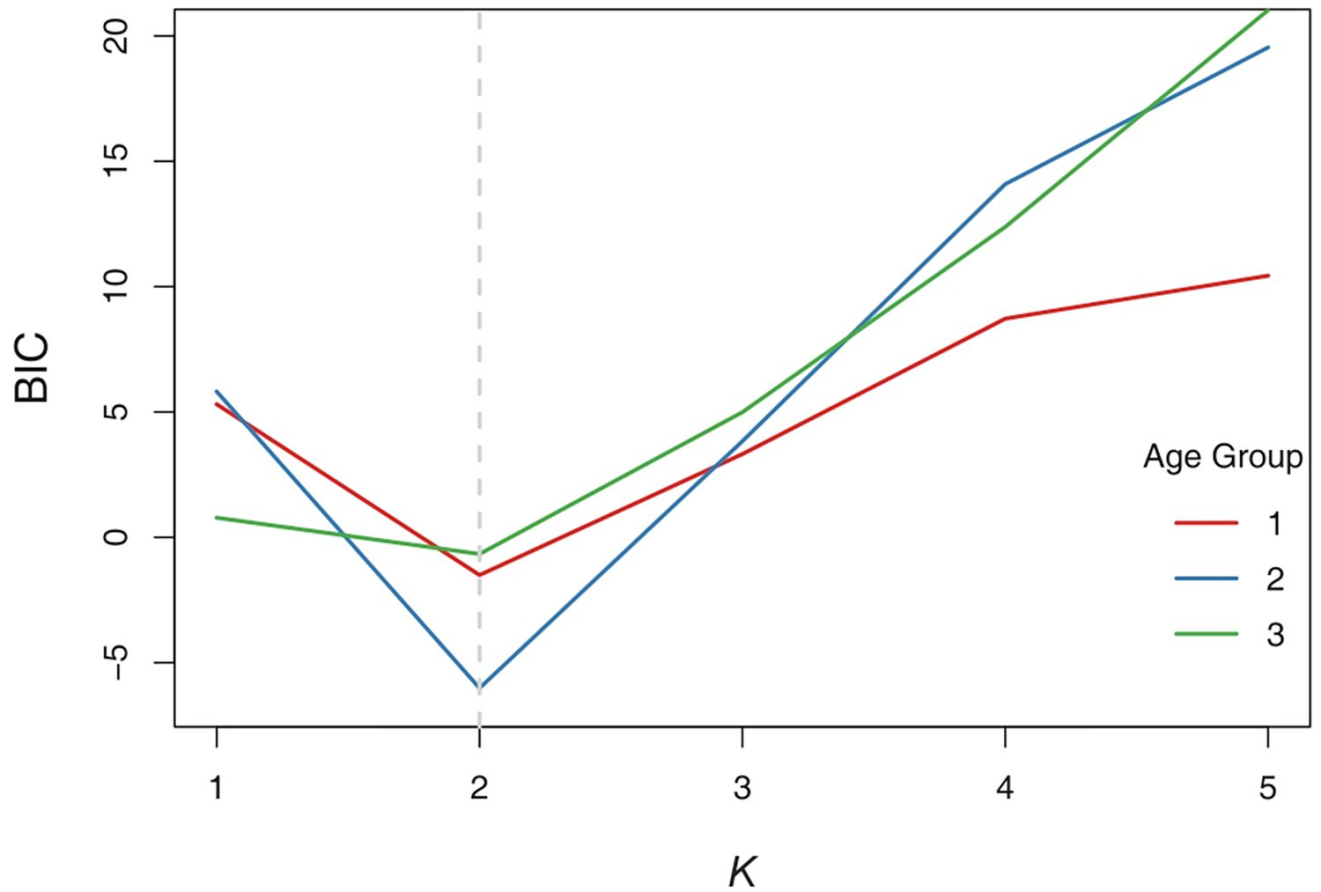
The BIC values corresponding to different choices of the number of components, *K*, in the mixture model of von Mises distributions fitted to circular data from 3 age-groups.

To demonstrate the focused structural diversity of RNFL loss, we applied finite mixture modeling, a popular technique for unsupervised learning of clusters, on the angular representations (as described above) of the glaucomatous eyes. In particular, we fitted a finite mixture of univariate von Mises distributions separately to 3 directional datasets on μ* directional values of glaucomatous eyes. First, we partitioned the cohort of glaucomatous eyes into 3 age-groups: Age1 (40–49 years), Age2 (50–59 years), and Age3 (> = 60 years). Then, each group’s dataset on μ* was modeled with a mixture of *G* ( = 1,2,…5) von Mises distributions with the aim of checking whether there are different clusters of glaucomatous eyes in these age-groups. Here, *G* = 1 implies a unimodal distribution of data that does not multiple distinct clusters. The models were fitted using the EM algorithm and the optimal model was selected based on the value of *G* for which BIC is the minimum (Fig 4).

**Fig 5:**
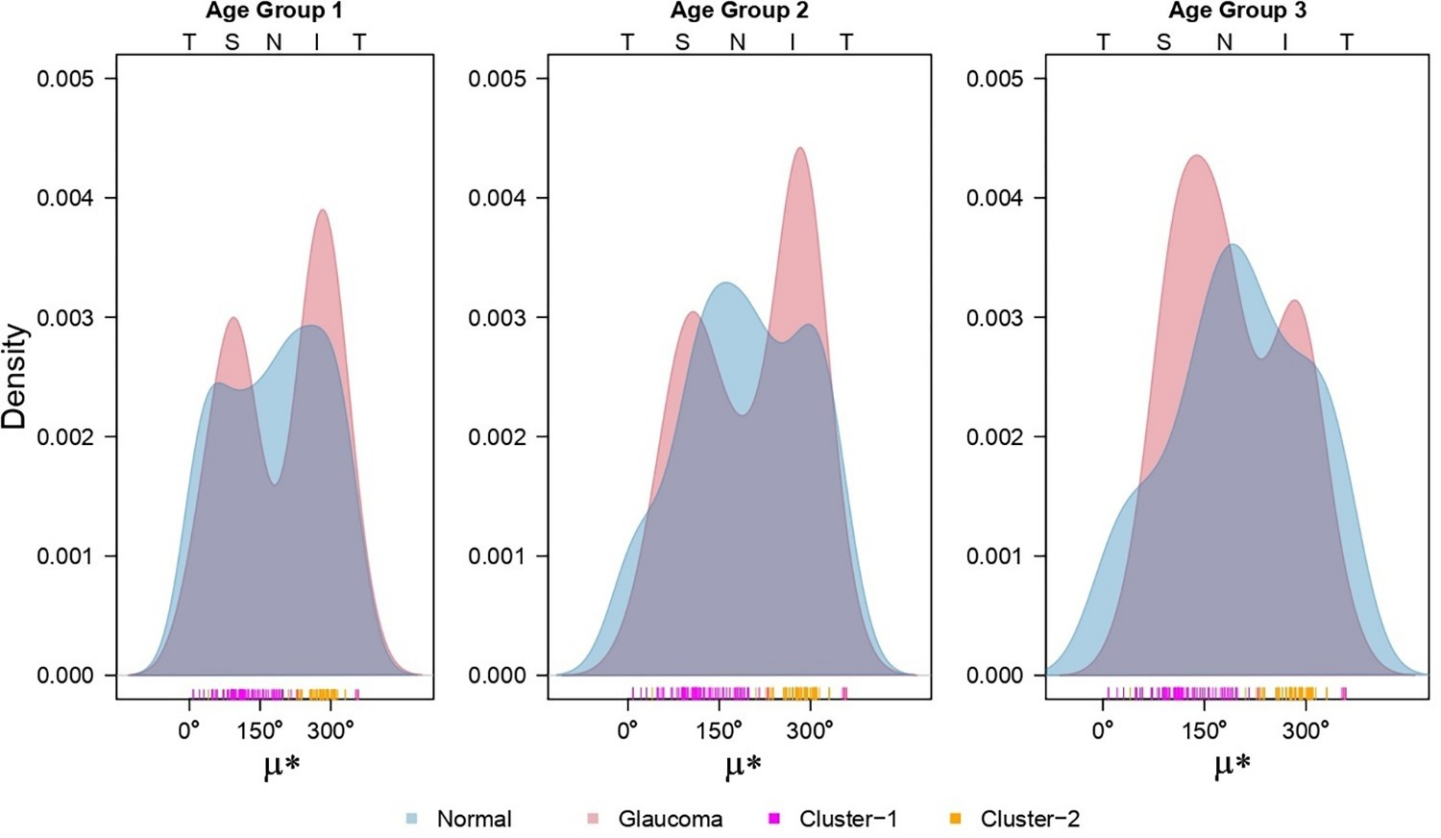
The distributions of circular data based on the angular representation of glaucomatous eyes from 3 age-groups are shown as red histograms in plots (a), (b) and (c). The corresponding normal distributions are shown as blue histograms. The assignment of the eyes to 2 clusters by the mixture model is shown as pink and orange points in the rugplot below the histograms. The TSNIT order of the circular range (0–360 degrees) is shown above the plots.

**Fig 6:**
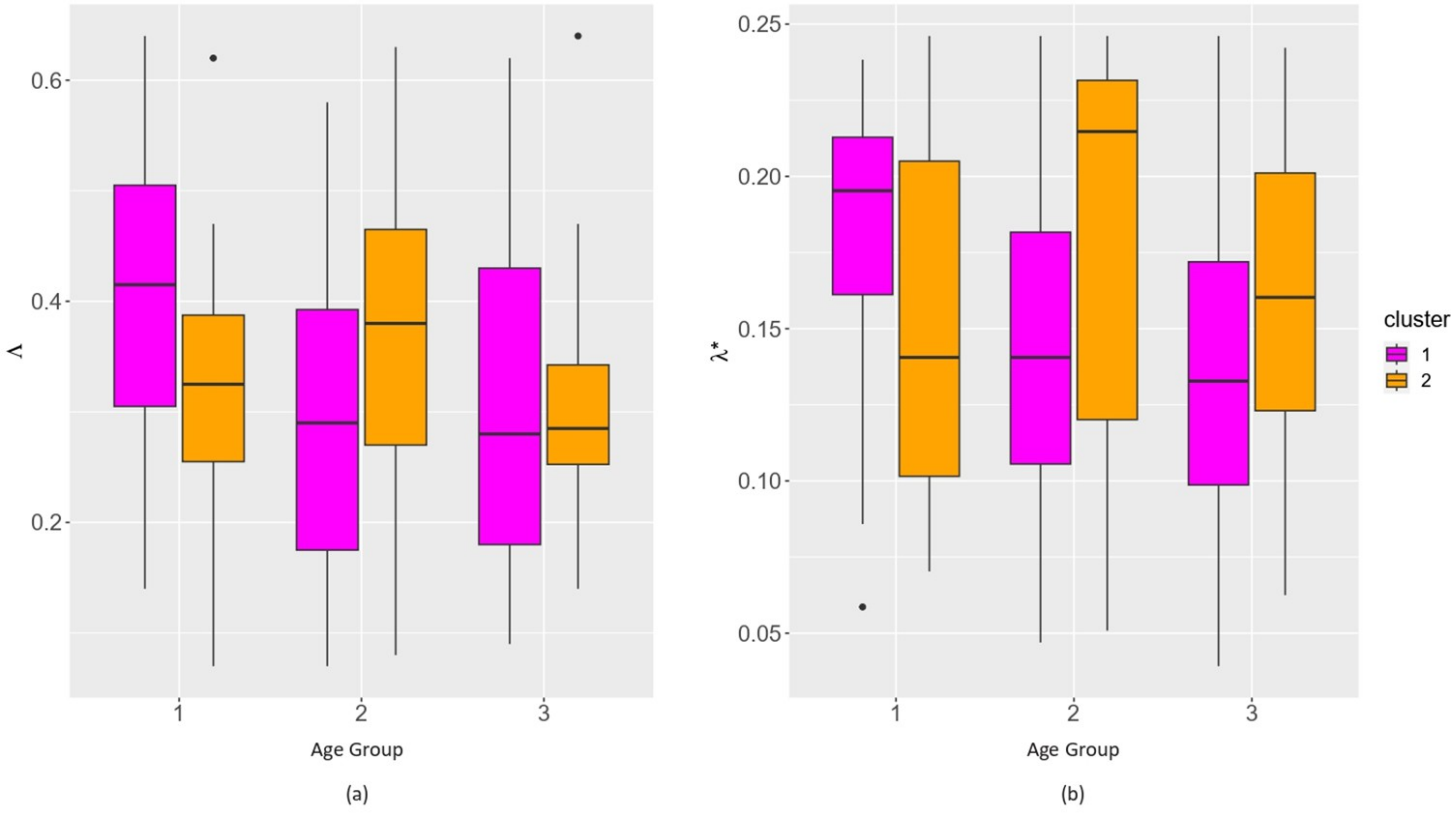
Boxplots comparing the values of the indices for (a) global and (b) local RNFL loss (y-axis) in the eyes belonging to 2 clusters in 3 age-groups (x-axis).

Interestingly, the optimally fitted von Mises mixture models identified 2 clusters (representing *G* = 2 mixture components) of glaucomatous eyes–in all 3 age-groups–with very distinct directional patterns of RNFL decay (red histograms in Fig 5). The estimated parameters of the selected models are shown in Table 2. In particular, we note that the 2 clusters align clearly across the 3 age-groups in terms of their very distinct estimates of the angular location parameter (*μ*) of the von Mises distribution that was fitted for identifying each component of the mixture. Relative to the higher RNFL thickness of the normal eyes in the Superior (S) and Inferior (I) sectors (see the green distribution in S1(B) Fig, the decay in the glaucomatous eyes is concentrated around these regions (the red “modes” in Fig 5) for all 3 age-groups. The rugplot at the bottom of the red histograms in Fig 6 depicts the membership of the glaucomatous eyes into these 2 clusters–using pink and orange points–as assigned by the fitted mixture model. In clear contrast to the glaucomatous eyes, the distributions of angular representation of RNFL data from the normal eyes of the same age groups appear much less distinctly multimodal in the blue histograms in Fig 5.

**Table 2:** The estimates of the parameters of a 2-component mixture model of von Mises distributions for 3 age-groups.

Group	Component	μ (degrees)	κ	α	BIC
Age1	Cluster1	101.97	11.044	0.304	-1.506
	Cluster2	296.26	1.556	0.696	
Age2	Cluster1	120.01	0.882	0.616	-6.015
	Cluster2	286.48	13.833	0.384	
Age3	Cluster1	146.45	1.247	0.784	-0.665
	Cluster2	291.77	14.932	0.216	

The Bayesian Information Criterion (BIC) value for each optimal model is shown.

For further characterization of the 2 clusters of glaucomatous eyes identified in different age-groups solely by their angular representation of RNFL decay, we studied their clinical covariates. In Table 3, we note that many of the clinical covariates of the glaucomatous eyes that belong to these 2 clusters have distinctive mean values. A visual comparison of such covariates across the 2 clusters in the 3 age-groups is shown in the boxplots of S2 Fig. For consistency, we used the same pink and orange color scheme for the corresponding clusters as in Fig 5. In Fig 6, upon comparison of the values of the new indices of global (Λ) and local loss (λ*) of RNFL, we again observed clear differences in the structural changes across the same clusters in different age-groups. Intriguingly, whereas the covariates and the global loss index could distinguish between the clusters in the glaucomatous eyes of the two younger groups, for the oldest group (Age3: ≥ 60 years), the distinction is pronounced only when we compared their indices of local RNFL loss λ*. We note that λ* clearly admits of higher values for the eyes in cluster 2 than those in cluster 1, which further underscores the utility of directional or focused analysis of OCT data.

**Table 3:** The mean values of the clinical covariates of the glaucomatous eyes belonging to the 2 identified clusters in 3 age-groups.

	Cluster1			Cluster2		
Variable (unit)	Age1	Age2	Age3	Age1	Age2	Age3
RIM AREA (mm^2)	1.09	1.09	1.13	1.02	1.09	1.05
DISC AREA (mm^2)	2.36	2.47	2.2	2.2	2.23	2.14
AVERAGE CD RATIO	0.72	0.72	0.68	0.7	0.68	0.7
AVERAGE THICKNESS (μm)	87.65	89.57	86.96	87.42	85.29	83.16
VERTICAL CD RATIO	0.68	0.69	0.66	0.69	0.69	0.69
CUP VOLUME (mm^3)	0.57	0.66	0.4	0.62	0.51	0.36
DISK DIAMETER (mm)	1.64	1.7	1.59	1.59	1.58	1.57

(Abbreviation: CD = cup to disc).

### Glaucoma classification using circular data

While the primary aim of the present study is to introduce circular representation and analysis of OCT data, we demonstrated the utility of our modeling of circular data to refine the classification of glaucomatous versus normal eyes. This was done in 2 steps: first, we built classification tree models based on variables that measure the global (Λ) and local (λ*) RNFL losses in each eye. We show these models in S3(A) and S3(B) Fig respectively for classification of samples in Age Group 3, which has the highest number of glaucoma samples. Interestingly, we noted that the majority of these samples (54.5% and 68.6%) were classified by the central leaf node of either tree with relatively high impurity (Gini index of 0.43 and 0.4). The overall model specificities are 0.762 and 0.761 respectively. In contrast, we used in step 2 the circular clustering labels obtained earlier (by von Mises mixture) to group the eyes first. Thus, we refined the classification of the same Age Group 3 with the tree model depicted in S3(C) Fig, which has higher specificity of 0.804, and has nodes of much less impurity (with maximum Gini index of 0.37).

## Discussion

While the investigation of localized RNFL defects in glaucoma diagnosis has been conducted in the past (e.g., [24], high-resolution data from the recent platforms allow us to approach this topic more systematically. SD-OCT has the ability to produce reproducible scans of an eye that could be read out as a high-resolution sequence of measurements made at a fairly large number (*N* = 256 in the present study) of evenly-spaced angular-points defined on a circular coordinate system. Yet, both of these insightful characteristics of OCT data–its granularity (*N*) and circularity–generally remain unutilized in its conventional downstream statistical analyses. Indeed, clinical applications may use results that are summarized over a much smaller number of angular sections, e.g., 4 quadrants or 12 clock-hours, and then too, the RNFL measures in these sections are utilized not for their directional information but only to capture the magnitude of loss therein. Hence, any comparison of OCT RNFL data–as is often done for patient classification or comparing across time-points–is generally restricted to the linear scale whereby no angle-specific distinction could be made among the focused losses.

In this study, we presented a new *circular statistical framework* that uses both the directionality as well as the magnitude of OCT RNFL thickness data. The framework could be straightforwardly generalized and applied to other comparable types of data such as OCT NRR, Fundus imaging, etc. In addition, we generated new high-resolution SD-OCT data. Using this input, an innovative output in the form of a directionally focused Angular Decay function was computed for each sample. Such a function provides a quantitative measure of RNFL loss–relative to the normal population–precisely at every 1.41 degree in a circular coordinate system. Notably, this allows for a well-defined data representation that takes values in the common range of (0,1) at a common sequence of angular-points around the circle with a fixed origin (0 degrees) for each sample and across all groups. Our standardized approach thus facilitates various downstream applications.

To begin with, we apply a tracing algorithm to identify the petals that are contiguous regions–defined as precisely specified angular intervals–at which the RNFL decay in a given eye exceeds a threshold that is specified with an intuitive quantile of the corresponding normal RNFL empirical distribution. To avoid a fixed choice of this threshold, our tracing algorithm uses a flexible parameter (*τ*). For instance, to focus on the last quartile of RNFL decay in our illustration, we used the value of *τ* = 0.75 although we understand that this choice is arbitrary. Importantly, based on the above framework, we introduced two measures of RNFL decay–both local (λ*) and global (Λ)–in a given eye relative to such decay in the normal eyes. In fact, we combined both the directionality (denoted by an angle) and the magnitude (by a weight) obtained from the OCT data in the form of a weighted circular mean to define a directionally focused *angular* representation (μ*) of a given eye’s RNFL decay.

The new representation opens up the potential for creative applications of the rich variety of tests and tools that exist in the fields of directional and circular statistics for insightful OCT data analysis. Such tools can provide novel insights into directionally focused segments of otherwise global phenomena such as RNFL loss in glaucoma patients. For instance, for the available left and right eye-pairs of the cohort in this study, the usual Pearson correlation of global RNFL loss (Λ) is 0.44. In contrast, the circular version of Pearson’s correlation [11] computed on the angular representation (μ*) of the same pairs of eyes is only 0.19, thus suggesting potential scope of circular statistics for expanding our capacity for finer differentiation of the clinical cohorts.

Interestingly, we demonstrated this capacity by modeling the structural heterogeneity in our cohort using a finite mixture of von Mises (also known as “circular normal”) distributions to the directional data generated in the present study. It led to unsupervised identification of 2 clusters with distinctive focused patterns of RNFL decay in 3 different age groups of a cohort of glaucomatous eyes from the Indian population. Moreover, the new indices of global and local RNFL loss were effective in capturing the structural differences between these glaucoma clusters. Furthermore, if the computed cluster membership of the eyes is used for grouping them first (i.e., according to the direction of focused decay), then it improved their (glaucoma versus normal) classification based on the local loss therein (as compared to conventional classification by global loss without circular clustering). These indices could be used either singly or in combination for downstream analysis of OCT data such as quantitative comparison of time-points or mining for new patterns of RNFL decay.

To accurately account for the effects of normal variation of RNFL thickness in a population, we emphasize that, ideally, normative databases of healthy eye OCT phenotypes that are large and racially representative should be developed. However, often such collections of healthy samples tend to be small or moderately sized, e.g., the normative database of Cirrus HD-OCT platform included just 284 subjects of which the representation of Indians is about 1% [16]. Clearly, this does not adequately reflect the 2020 projections about India which is to become the second in global glaucoma numbers, surpassing Europe [4]. In this direction, our rigorous cataloging of the more than 2300 directionally precise distributions of RNFL thickness, which takes into account the variations of age and optic disc sizes in the normal population, provides a unique quantitative reference for the focused angle-specific decay in the glaucomatous eyes. Such population-level insights could be helpful in setting appropriate baselines for clinical decision-making. Importantly, the catalog contributes to addressing the current disparity in representation of Asian Indian RNFL structural phenotypes in the normative OCT databases.

We understand that there are certain limitations of the study. While our adaptation of the image processing algorithm, as implemented in the standard Cirrus HD-OCT software package, for generating the *N* = 256 point circular RNFL data is straightforward, we understand that such high-resolution data extraction is not common in OCT analysis. Moreover, the focused and angular measure of the new directional representation of RNFL loss renders it difficult to conduct a direct comparison with any traditional indices that are based on the magnitude (but not directionality) of such decay. Finally, we identified, in 3 different age-groups, 2 recurrent clusters of glaucomatous eyes with very distinct directional signatures of RNFL decay. In future work, we will provide further characterization of the clusters with more rigorous investigation that goes beyond the scope of the present study.

While past studies have used spatial, trigonometric and Fourier analysis on OCT data, e.g., [25–28], our framework, involving directional or circular data, is methodologically quite different from those approaches. While our earlier platform CIFU (14) focuses on the aspects of shapes and curves in OCT NRR data, as in functional data analysis (FDA), it does not explicitly address the directional characteristics of OCT data or angular measurements thereof. We have demonstrated the utility of both the granularity and the directionality of OCT data and combined these with an angle-based description of patterns that could be studied with innovative downstream analyses. Indeed, processes or platforms that generate data which are capable of being represented as angular values (e.g., wind direction, neuronal activity) require altogether different statistical approaches as compared to usual linear data [10]. We think that the new directional representation of OCT data to capture an eye’s RNFL decay will pave the way for future applications of the rich methodology of directional and circular statistics [10–12] to eye data analysis.

## Supporting information

S1 Fig (a) RNFL Deviation Map: The OCT device measures the RNFL thickness throughout the 200 × 200 data cube, where each A-scan represents a pixel corresponding to a 30 μm wide square. The Bruch’s membrane opening (BMO) circle (black), cup border (red) and RNFL calculation circle (purple) are superimposed on the OCT’s enface infrared image. (b) RNFL Thickness TSNIT Plot: Cirrus HD-OCT software extracts a sequence (black curves for OD/OS) of N = 256 data points (y-axis) from the 200 × 200 Disc Cube area for the calculation circle (x-axis). The color-coded background represents the ranges of RNFL thickness based on the observations in the manufacturer’s age-matched normative database. Note, as the y-axis does not depict relative frequency of the observations, it is not a circular density function.
(JPG)

S2 Fig Boxplots of clinical covariates (y-axis) of glaucomatous eyes belonging to the 2 clusters identified by the von Mises mixture model. Three age-groups are shown in the x-axis.
(JPG)

S3 Fig Decision tree models for classification of normal vs. glaucomatous eyes using variables for (a) global loss without circular data, (b) local loss without circular data, and (c) local loss with partitioning of the data by circular cluster means. The significance of splitting a node of the tree by the selected variable is given by a p-value.
(JPG)

S1 Table High-resolution OCT RNFL thickness dataset for normal Asian Indian eyes. Each row represents an anonymized normal eye. Each column represents an angular point on the circle.
(CSV)

S2 Table High-resolution OCT RNFL thickness dataset for glaucomatous Asian Indian eyes. Each row represents an anonymized glaucomatous eye. Each column represents an angular point on the circle.
(CSV)

S3 Table The deciles of RNFL thickness at each angular-point for a cohort of normal eyes stratified by 3 age groups (appears in 3 separate tabs in the spreadsheet) and 3-disc sizes (appears with 3 headings). (XLSX)
