# Supplementary figures and images for: Focused analysis of RNFL decay in glaucomatous eyes using circular statistics on high-resolution OCT data
Source: PLoS One. 2023 Oct 18;18(10):e0292915. doi: 10.1371/journal.pone.0292915 (PMC10584101; doi:10.1371/journal.pone.0292915)

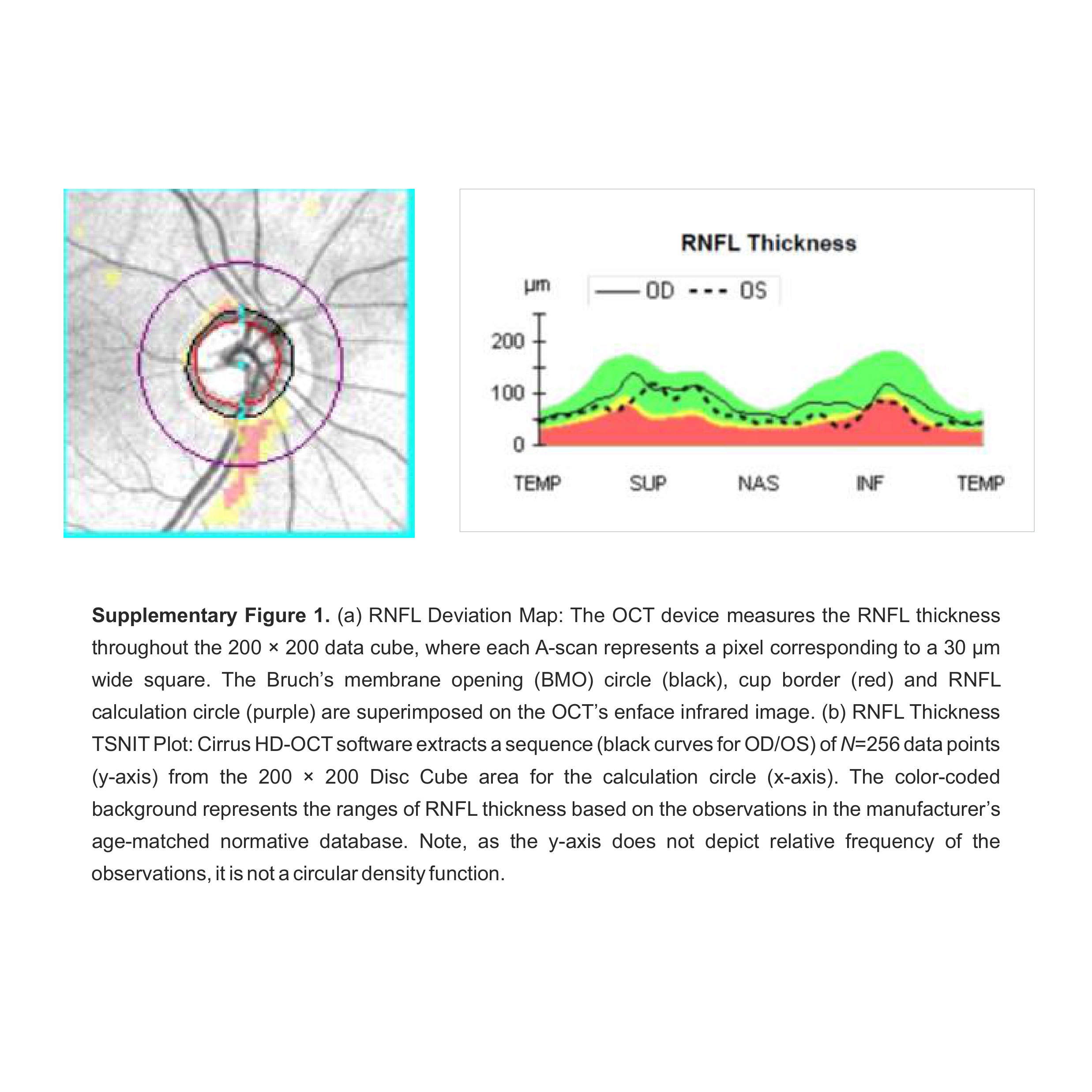

Supplement: S1 Fig — (a) RNFL Deviation Map: The OCT device measures the RNFL thickness throughout the 200 × 200 data cube, where each A-scan represents a pixel corresponding to a 30 μm wide square. The Bruch’s membrane opening (BMO) circle (black), cup border (red) and RNFL calculation circle (purple) are superimposed on the OCT’s enface infrared image. (b) RNFL Thickness TSNIT Plot: Cirrus HD-OCT software extracts a sequence (black curves for OD/OS) of N = 256 data points (y-axis) from the 200 × 200 Disc Cube area for the calculation circle (x-axis). The color-coded background represents the ranges of RNFL thickness based on the observations in the manufacturer’s age-matched normative database. Note, as the y-axis does not depict relative frequency of the observations, it is not a circular density function. (JPG) [file pone.0292915.s001.jpg]

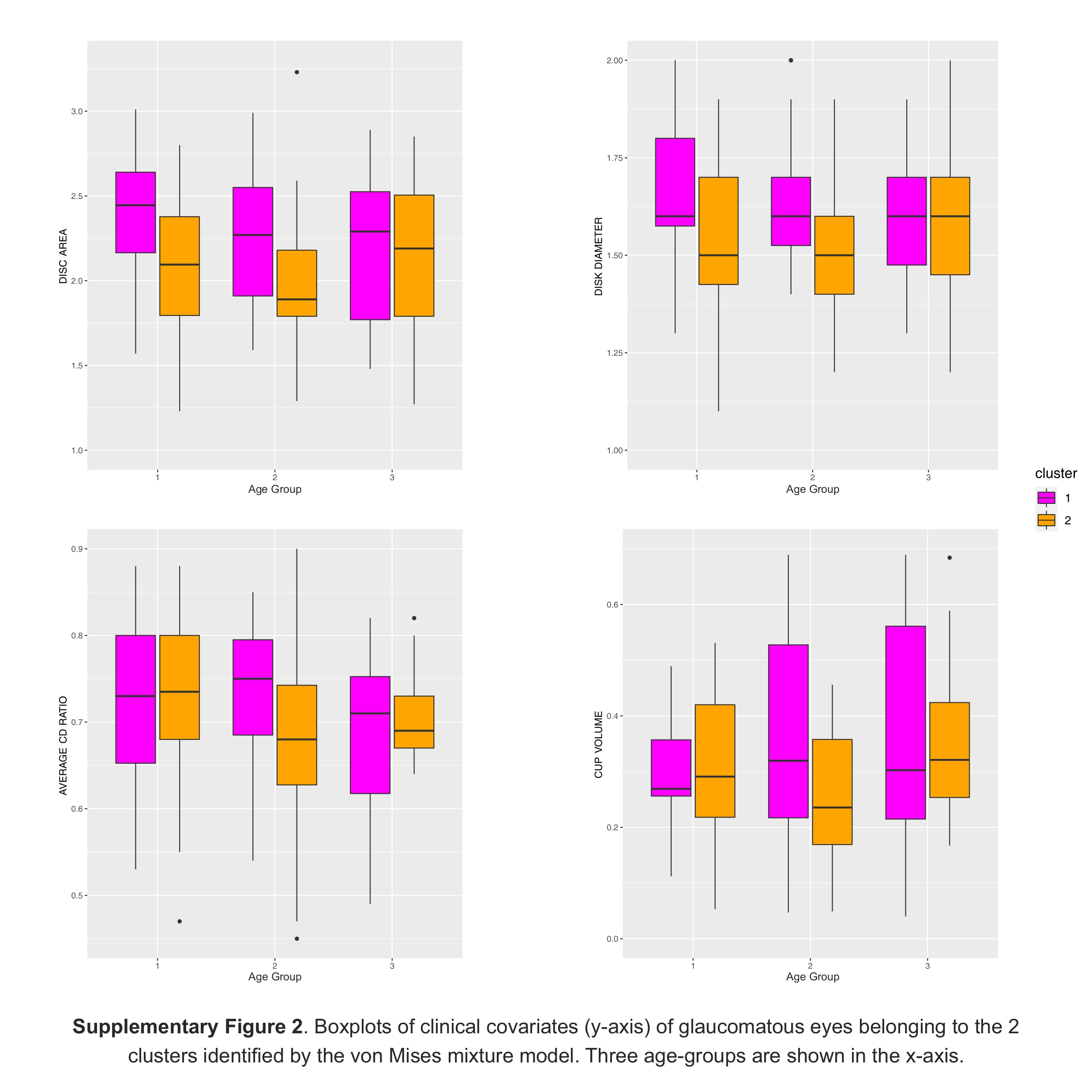

Supplement: S2 Fig — Three age-groups are shown in the x-axis. (JPG) [file pone.0292915.s002.jpg]

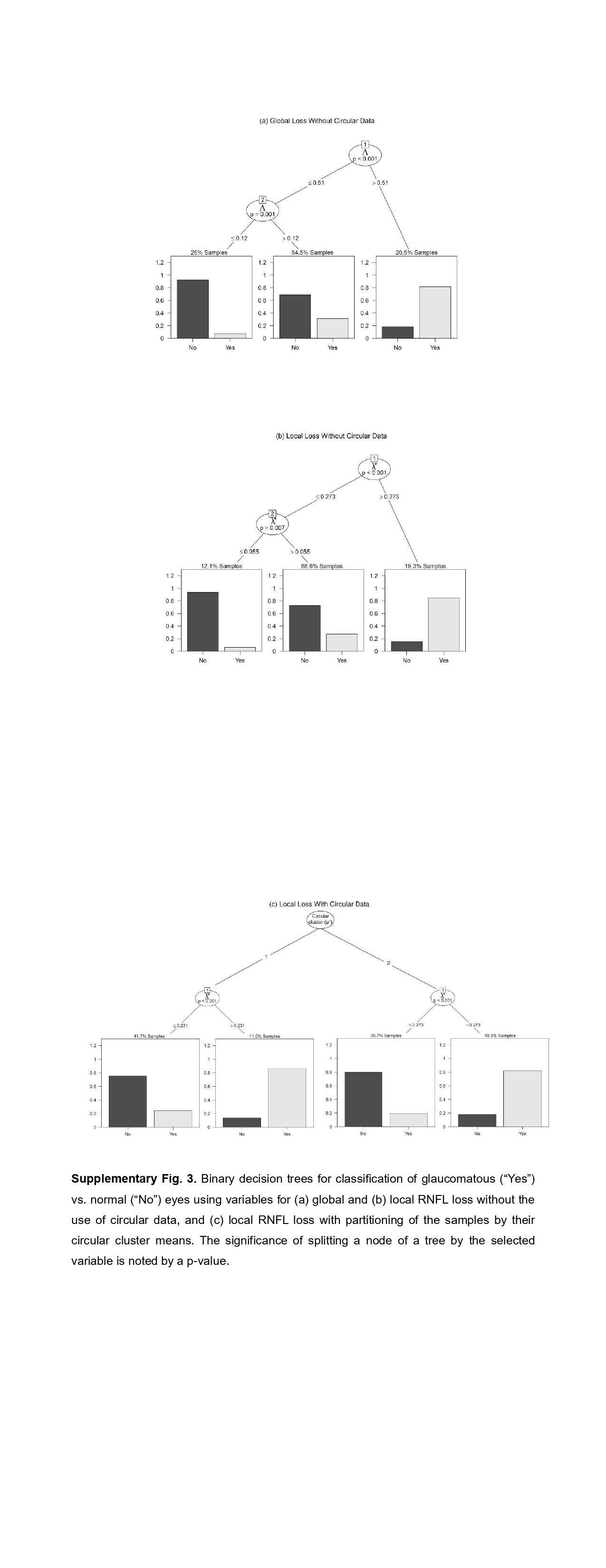

Supplement: S3 Fig — Decision tree models for classification of normal vs. glaucomatous eyes using variables for (a) global loss without circular data, (b) local loss without circular data, and (c) local loss with partitioning of the data by circular cluster means. The significance of splitting a node of the tree by the selected variable is given by a p-value. (JPG) [file pone.0292915.s003.jpg]
